# Loss of TGFβ signaling promotes colon cancer progression and tumor-associated inflammation

**DOI:** 10.18632/oncotarget.9830

**Published:** 2016-06-04

**Authors:** Daniel R. Principe, Brian DeCant, Jonas Staudacher, Dominic Vitello, Riley J. Mangan, Elizabeth A. Wayne, Emman Mascariñas, Andrew M. Diaz, Jessica Bauer, Ronald D. McKinney, Khashayarsha Khazaie, Boris Pasche, David W. Dawson, Hidayatullah G. Munshi, Paul J. Grippo, Barbara Jung

**Affiliations:** ^1^ University of Illinois College of Medicine, Urbana-Champaign, IL, USA; ^2^ Department of Medicine, University of Illinois at Chicago, Chicago, IL, USA; ^3^ Robert H. Lurie Comprehensive Cancer Center, Feinberg School of Medicine, Northwestern University, Chicago, IL, USA; ^4^ Department of Immunology, Mayo Clinic College of Medicine, Mayo Clinic, Rochester, MN, USA; ^5^ Comprehensive Cancer Center of Wake Forest University, Winston-Salem, NC, USA; ^6^ Department of Pathology and Laboratory Medicine, Jonsson Comprehensive Cancer Center, David Geffen School of Medicine at UCLA, Los Angeles, CA, USA; ^7^ Department of Medicine, Northwestern University, Chicago, IL, USA

**Keywords:** colon cancer, Inflammation, TGF-beta

## Abstract

TGFβ has both tumor suppressive and tumor promoting effects in colon cancer. Also, TGFβ can affect the extent and composition of inflammatory cells present in tumors, contextually promoting and inhibiting inflammation. While colon tumors display intratumoral inflammation, the contributions of TGFβ to this process are poorly understood. In human patients, we found that epithelial loss of TGFβ signaling was associated with increased inflammatory burden; yet overexpression of TGFβ was also associated with increased inflammation. These findings were recapitulated in mutant APC models of murine tumorigenesis, where epithelial truncation of TGFBR2 led to lethal inflammatory disease and invasive colon cancer, mediated by IL8 and TGFβ1. Interestingly, mutant APC mice with global suppression of TGFβ signals displayed an intermediate phenotype, presenting with an overall increase in IL8-mediated inflammation and accelerated tumor formation, yet with a longer latency to the onset of disease observed in mice with epithelial TGFBR-deficiency. These results suggest that the loss of TGFβ signaling, particularly in colon epithelial cells, elicits a strong inflammatory response and promotes tumor progression. This implies that treating colon cancer patients with TGFβ inhibitors may result in a worse outcome by enhancing inflammatory responses.

## INTRODUCTION

While the prognosis of colon cancer is generally favorable when diagnosed at early stages, advanced disease is associated with a high mortality, and colon cancer remains one of the main contributors of cancer-related deaths in developed countries. Recent data suggests that, while early stage incidence is declining with intensified screening, the incidence of late stage colon cancer is in fact increasing, particularly in young adults [1]. Emerging evidence confirms marked survival differences based on molecular subtypes of colon cancers [2], and there remains an unmet need for effective, individualized treatments for patients with late stage disease.

To this end, Transforming Growth Factor β (TGFβ) is known to play several crucial roles in colon carcinogenesis. While canonical SMAD-mediated TGFβ signaling is largely tumor suppressive [3–5], recent evidence implicates TGFβ as a key pathway in the metastatic progression of colon cancer [6–8]. Hence, pharmacological inhibition of TGFβ signals or their receptors (TGFBRs) has been suggested as a potential therapeutic approach in advanced colon cancer patients. However, the effects of TGFβ in the tumor microenvironment include both pro- and anti-carcinogenic events that are highly varied and still poorly understood.

The role of TGFβ as a tumor suppressor is substantiated by previous work identifying epithelial loss of SMAD4, indicative of disrupted TGFβ signaling, in the progression of sporadic microsatellite stable advanced colorectal tumors [5]. Interestingly, the other common genomic subtype, microsatellite instability (MSI) in colon cancers, often present with mutations in TGFβ receptors, a better prognosis [9], and a CD8^+^ rich immune infiltrate likely associated with targetable immune checkpoints [10].

The immune system is a well-known contributor to the onset and progression of many malignancies including colon cancer. Colon tumors display robust intratumoral inflammation, involving both lymphoid and myeloid cells, namely macrophages, which are a predominant source of cytokines that have been demonstrated to affect cancer cell proliferation and migration [11, 12]. Such cytokines, including TGFβ, can affect the extent and composition of inflammatory cells present in tumors. For example, TGFβ can convert CD4^+^ T helper cells to suppressive CD4^+^FoxP3^+^ regulatory T cells (Tregs), which in turn inhibit cell-mediated immunity via the release of suppressive factors such as IL10 [13]. However, TGFβ can also act in concert with IL6 to direct the T-helper 17 (Th17) differentiation program, leading to the attraction and activation of inflammatory granulocytes such as neutrophils and macrophages [14], thereby promoting the inflammatory response. In addition, TGFβ signaling contributes to myeloid cell function, particularly macrophages. Mice with conditional deletion of TGFBR2 in the bone marrow presented with fewer anti-inflammatory M2 macrophages and a lethal inflammatory phenotype. This study further demonstrated that ex vivo TGFBR2-null bone marrow derived macrophages have impaired M2 polarization, thereby implicating TGFβ in macrophage function [15].

Given the multitude of functions of TGFβ on epithelium and the tumor microenvironment, we first determined the relationship between TGFβ signaling and tumoral myeloid infiltration in colon cancer patients. We next examined the effects of diminished TGFβ signaling, both globally and restricted to the epithelium, on murine colon cancer development. Using a mutant APC mouse model of colon cancer, we demonstrate that both epithelial and global suppression of TGFβ signaling are associated with a robust inflammatory response in the colon, mediated predominantly by myeloid cells. This response is highly augmented in the more physiologically relevant model of epithelial TGFβ signaling deficiency, which developed lethal disease and severe weight loss. Overall, our findings demonstrate that loss of TGFβ signaling, particularly when confined to epithelial cells as it commonly occurs in colon cancer, worsens tumor progression and outcome. Thus our findings call for detailed risk stratification in advanced colon cancer patients prior to the consideration of TGFβ-inhibition therapy.

## RESULTS

### Loss of SMAD4 and TGFβ overexpression correlate with increased myeloid infiltration in colon cancer patients

**Figure 1:**
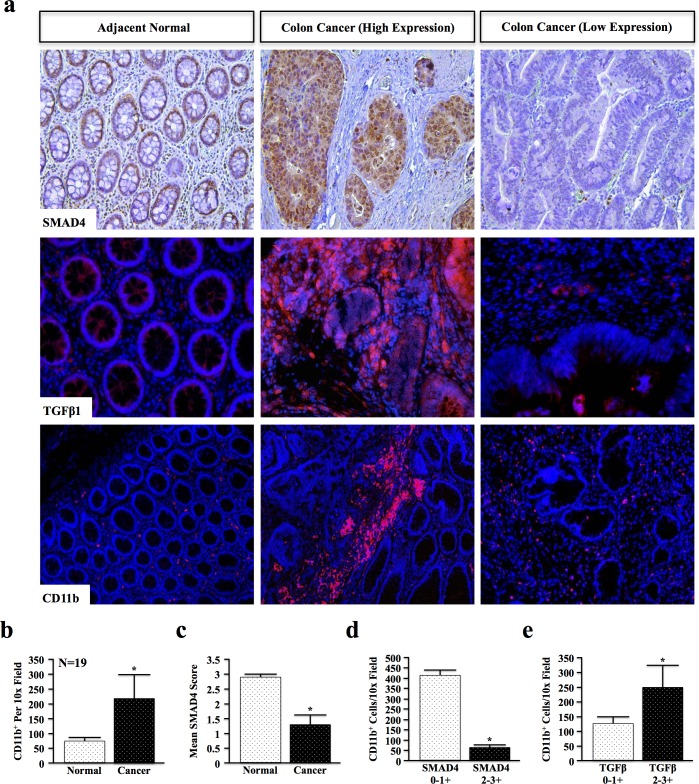
Loss of SMAD4 and TGFβ Overexpression Correlate with Increased Myeloid Infiltration in Colon Cancer Patients **a.** Normal and cancer tissue from human colon cancer patients were stained for SMAD4, TGFβ1 and myeloid marker CD11b. SMAD4 and TGFβ1 staining intensity were blindly scored by two investigators from 0-3+ (representative images shown for low (0), intermediate (1+), and high (3+)), and CD11b+ cells quantified per 10x field. **b.** Quantification of CD11b+ revealed an increase in myeloid cell involvement in cancer when compared to adjacent normal tissue, consistent with increased inflammation. **c.** The mean SMAD4 score was reduced in colon cancer tissue (1.33) compared to normal sections (2.89). **d.** SMAD4 low patients (scores of either 0 or 1+) had significantly higher CD11b+ populations than SMAD4 intact (2 or 3+) populations. e. Patients with elevated TGFβ1 expression in the colon microenvironment displayed increased CD11b+ cell involvement. (*, P < 0.05).

As TGFβ may display both pro- and anti-inflammatory effects, we first examined the relationship between TGFβ signaling and inflammation in genomically unidentified human colon cancer specimens. Colon cancer and adjacent normal sections were stained for SMAD4, TGFβ1, and CD11b, a myeloid lineage marker expressed by tumor-associated macrophages, neutrophils, and other inflammatory cells (Figure 1a and Table S1, N = 19). SMAD4 and TGFβ1 were independently scored from 0-3+ based on staining intensity by two blinded investigators, and CD11b^+^ cells counted per a lower power (10x) field to more accurately determine the number of infiltrating cells. Criteria for scoring are detailed in Supplementary Table S2.

Quantification of CD11b^+^ cells revealed a near 3-fold increase in the average number of myeloid cells in cancer compared to adjacent normal tissue (Figure 1b). Additionally, epithelial expression of SMAD4 was decreased by nearly 50% in cancer specimens compared to adjacent normal tissue (Figure 1c and Table S1). Moreover, patients with low (0-1+) SMAD4 expression had significantly higher CD11b^+^ infiltration, consistent with increased inflammation, than patients with high (2-3+) SMAD4 expression (Figure 1d). However, CD11b^+^ infiltration was also increased in patients with high expression of the TGFβ1 ligand (Figure 1e) that was not statistically related to SMAD4 status (data not shown). Combined, these data suggest that the effects of epithelial TGFβ signaling and the overall effects in the tumor microenvironment on inflammation may in fact be highly varied.

### TGFBR-deficiency accelerates murine intestinal tumorigenesis

**Figure 2:**
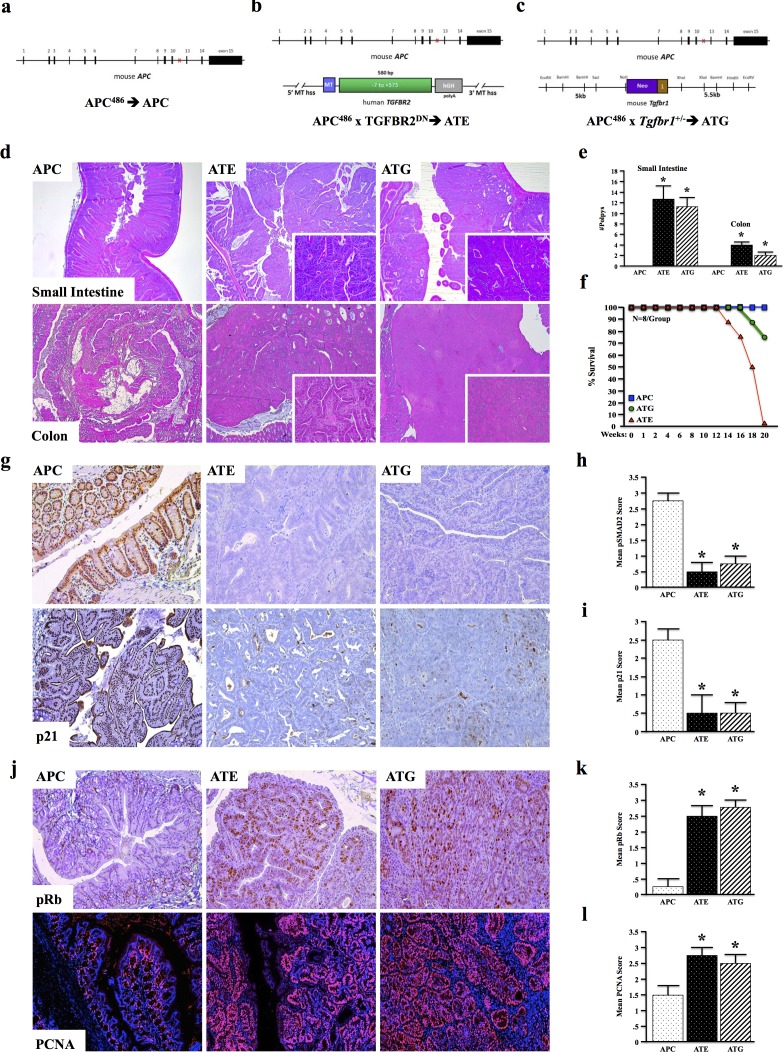
TGFBR-Deficiency Accelerates Murine Intestinal Tumorigenesis **a.** APCΔ468 (APC) mice were generated as a model of pre-neoplastic disease of the intestinal tract. **b.** APC mice were crossed to MT-TGFBR2DN (TE) mice to generate a model of epithelial TGFβ signaling deficiency (ATE mice). **c.** APC mice were crossed to mice with global, heterozygous deletion of Tgfbr1 (TG) to form ATG mice. d. At four months, APC mice displayed mild dysplasia in both the colon and small intestine. However, ATE mice presented with several large tubular adenomas, some of which obstructed the lumen of the colon/small intestine. Also at four months, ATG mice presented with large tubular adenomas of the colon. **e.-f.** Consistent with loss of epithelial TGFβ signaling, ATG and ATE mice both displayed loss of pSMAD2. **g.-j.** ATE and ATG mice also presented with increased pRB and PCNA staining, consistent with loss of epithelial cell cycle arrest. (*, P < 0.05. N = 4 per group).

To further understand the overall role of TGFβ signaling in colon cancer development and tumor associated inflammation in vivo, we crossed APC^Δ468^ (APC) mice (Figure 2a), a model of intestinal neoplastic disease, to those expressing a metallothionein-driven dominant-negative TGFBR2 (MT-TGFBR2^DN^ or TE) to provide a tumor model with strictly epithelial [16] suppression of TGFβ signaling (ATE) (Figure 2b). This model faithfully recapitulates the TGFBR2 inactivating mutations commonly observed in the clinic, and allows for TGFβ signaling in the entire gastrointestinal tract. However, as TGFβ has been shown to drive the progression of colon cancer via its effects in the tumor microenvironment, we bred APC to those with global TGFβ signaling deficiency (Tgfbr1^+/−^ or TG) to form ATG, which more closely mimics the effects of systemic administration of a TGFβ inhibitor, the majority of which target TGFBR1 (Figure 2c).

While APC mice displayed only mild dysplasia in both the colon and small intestine at four months, ATE mice presented with more pronounced loss of tissue architecture and accelerated polyp formation in both the small intestine and colon (N = 8). These mice developed several large tubular adenomas, some of which obstructed the lumen of the colon/small intestine (Figure 2d, 2e). Additionally, 2/8 ATE mice presented with detached fragments of adenomatous tissue and locally invasive cancer. ATG mice (N = 8) similarly developed multiple tubular adenomas and prominent luminal masses within four months, both in the colon and the small intestine (Figure 2d, 2e). While 100% of APC mice survived 20 weeks, 100% of ATE mice died between 14 and 20 weeks, presumably by catabolic wasting. In contrast, 75% of ATG animals survived 20 weeks, with death only occurring in 2/8 mice, between 18 and 20 weeks (Figure 2f). Despite these differing phenotypes, both ATE and ATG had decreased epithelial staining for pSMAD2 and p21 (Figure 2g-2i) with increased pRb and PCNA staining (Figure 2j-2m), all consistent with the loss of TGFβ-induced cell cycle arrest.

### Both systemic and epithelial TGFBR-deficiency enhance APC-induced inflammation

**Figure 3:**
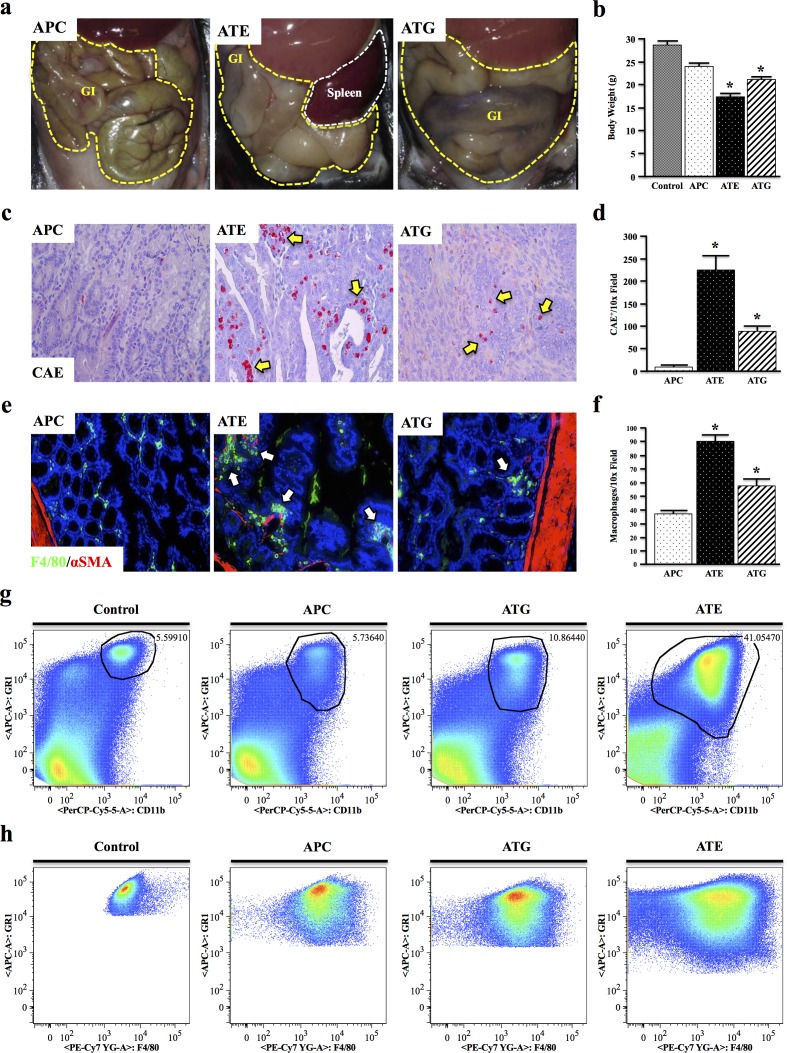
Both Systemic and Epithelial TGFBR-Deficiency Enhance APC-Induced Inflammation **a.** At four months, ATE and ATG mice presented with severely distended GI tracts compared to APC mice. **b.** ATE and ATG mice were also grossly underweight, weighing on average ~60% of control mice. **c.-d.** Mouse tissues were subject to Chloracetate Esterase (CAE) staining, which indicated a near 26-fold increase in the number of inflammatory granulocytes in the colon of ATE mice compared to APC controls, and a near 2.5-fold increase compared to ATG. **e.-f.** Staining for the mouse macrophage marker F4/80, which showed an increase in tumor infiltrating macrophages in TGFBR-deficient mice, most notably in ATE, localizing predominantly to the colon stroma. **g.** Spleens were harvested from nongenic control, APC, ATG, and ATE animals, and analyzed for expression of the myeloid marker CD11b and GR1, a marker of most inflammatory granulocytes. While ATG animals displayed an approximate two-fold increase in GR1+CD11b+ cells, ATE mice had a 7.33-fold increase over control mice, consistent with a more severe inflammatory phenotype. **h.** Gating to GR1+CD11b+ populations in control and mouse models revealed strong positivity for the macrophage marker F4/80. (*, P < 0.05. N = 4 per group). It should be noted that, due to the large differences in the size and location of the population of interest, it was necessary to gate manually.

At four months of age, while the gastrointestinal tract of APC mice was fairly normal, those of both ATE and ATG mice were severely distended, being most dramatic in ATE mice (Figure 3a, N = 8 per group). ATE mice were also grossly underweight, with a body weight ~40% lower than that of control mice, and presented with pronounced rectal bleeding and prolapse (data not shown). ATG mice were also significantly underweight, though to a lesser extent (Figure 3b).

To determine if there is an inflammatory component to these phenotypes, colon sections were subject to Chloracetate Esterase (CAE) staining, revealing a significant increase in inflammatory mast cells in the neoplastic tissues of ATG, and particularly ATE mice compared to the more normal mucosa of APC mice (Figure 3c, 3d). These results were corroborated by staining for the mouse macrophage marker F4/80, which showed an increase in macrophage infiltration in TGFBR-deficient mice, localizing predominantly to the stroma of normal mucosa adjacent to the neoplastic events (Figure 3e, 3f). Interestingly, there was no change in macrophage polarization in all three models (Figure S1). To exclude the possibility of ectopic TGFβ inactivation in the leukocytes of ATE mice, tissue sections were dual-stained for pSMAD2 expression in T cells and macrophages, respectively (Figure S2 and Figure S3). Furthermore no gross inflammatory changes were observed in other tissues (data not shown).

While ATG mice displayed a slight increase in splenic CD11b^+^GR1^Hi^ inflammatory granulocytes compared to control and APC mice, ATE mice showed a near 8-fold and 4-fold increase compared to APC and ATG mice, respectively (Figure 3g). Additionally, gating to CD11b^+^GR1^Hi^ cells revealed these cells were highly positive for F4/80, which increased incrementally in APC, ATG, and ATE mice, respectively (Figure 3h) and is likely indicative of the increased expansion/recruitment of splenic macrophages in ATG and ATE groups (N = 3 per group).

### TGFBR-deficiency modifies cancer associated cytokines *in vivo*

**Figure 4:**
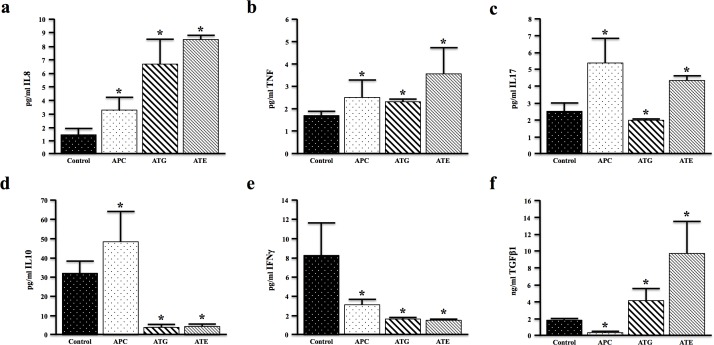
TGFBR-Deficiency Modifies Cancer Associated Cytokines In Vivo. **a.-c.** Serum from control (nongenic), APC, ATG, and ATE mice was evaluated for expression of the pro-inflammatory cytokines IL8, TNFα, and IL17. IL8 was increased in both ATG and ATE mice, though TNFα, and IL17 were only significantly elevated in ATE mice. **d.-e.** Serum levels of the anti-inflammatory cytokine IL10 and interferon-γ (IFNγ) were reduced in both ATG and ATE mice again consistent with an increased inflammatory phenotype in TGFBR-deficient mice. **f.** Additionally, levels of serum TGFβ1 were significantly higher in ATE mice when compared to all other cohorts. (*, P < 0.05. N = 4 per group).

To understand the upstream difference in the extent of inflammation in the described models of TGFβ signaling deficiency, we examined the serum levels of cytokines commonly implicated in colon cancer development. Both ATG and ATE mice displayed increased serum levels of IL8 (the mouse IL8 analogue, KC) compared to nongenic and APC controls (Figure 4a). However, the pro-inflammatory cytokine TNFα was noticeably increased in ATE mice (Figure 4b). Interestingly, globally TGFBR-deficient ATG mice showed reduced serum levels of the pro-inflammatory, Th17 derived cytokine IL17, which was elevated in APC and ATE (Figure 4c). In addition, both ATG and ATE mice demonstrated significantly lower serum levels of the anti-inflammatory cytokine IL10 (Figure 4d) and interferon-γ (IFNγ) (Figure 4e). In contrast, both ATG and ATE mice demonstrated increased levels of TGFβ1 (Figure 4f), with levels of TGFβ1 dramatically increased in ATE mice. The comparatively low levels of TGFβ in the ATG cohort may be a function of the colon stroma, as TGFβ induces its own expression through these cells (Figure S4). However, taken together, these observations are consistent with an increased inflammatory phenotype in ATG, and specifically ATE animals compared to TGFBR-intact controls.

### Systemic TGFBR-deficiency exacerbates conditional APC^Δ468^ driven colon tumorigenesis

While we have demonstrated that either epithelial or systemic TGFβ signaling deficiency accelerates intestinal tumorigenesis in an inherited model of mutant APC^Δ468^, global APC mutations are representative of a small subgroup of cancer patients, the overwhelming majority of which present with sporadic APC mutations [17]. Therefore, to assess the effects of global TGFβ signaling deficiency in a more clinically relevant model of sporadic colon cancer, TS4-Cre mice were crossed to those with a floxed APC^Δ468^ construct (Ts4-Cre/APC^fl/fl^ or cAPC) to restrict mutant APC expression to the colon, allowing for a more faithful recapitulation of APC mutations as they most often present in the clinic.

**Figure 5:**
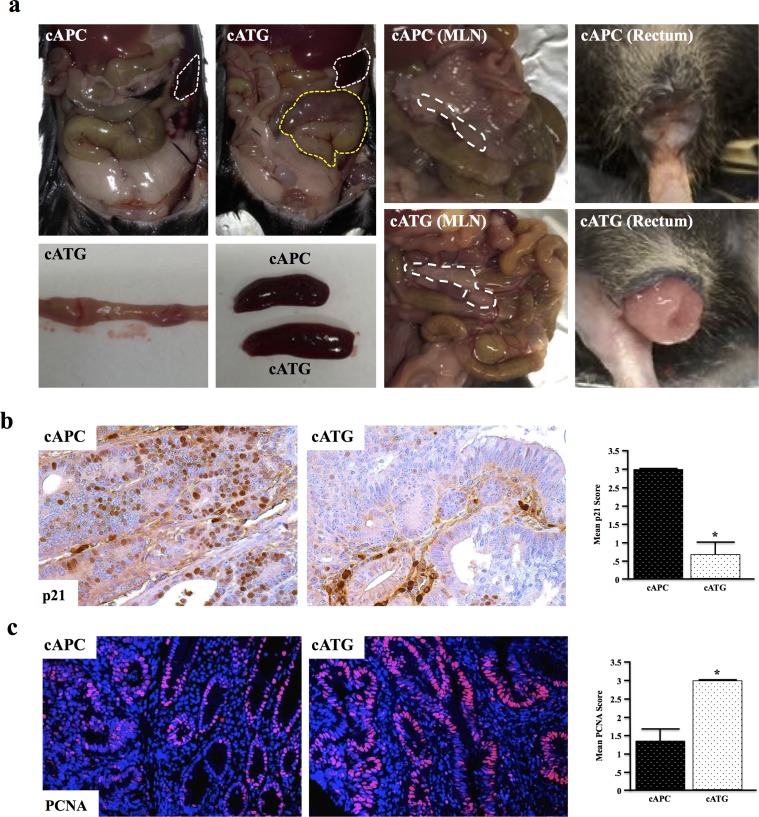
Systemic TGFBR-Deficiency Exacerbates Conditional APC Δ468 Driven Colon Tumorigenesis **a.** Ts4-Cre/APCfl/fl (cAPC) mice were generated and crossed to Tgfbr1+/− (TG) mice to form cATG. At four months, cAPC mice displayed mild dysplasia exclusively in the colon, with only 1/8 mice developing adenomas/polyps. However, 100% of cATG mice presented with large tubular adenomas in the colon visible from outside the organ. cATG mice also displayed gross splenomegaly, as well as lymphadenopathy and rectal prolapse. **b.,c.** Tissue sections were stained with anti-p21 and anti-PCNA respectively to assess changes in TGFβ-induced cell cycle arrest. (*, P < 0.05. N = 4 per group).

At four months, cAPC mice present with mild distention of the gastrointestinal tract, though are otherwise normal. cAPC mice were next crossed to mice with global TGFβ signaling deficiency (Tgfbr1^+/−^ or TG) to form cATG mice. At four months, cATG mice presented with large tubular adenomas visible from outside the colon. These mice also developed gross splenomegaly, mesenteric lymphadenopathy, and rectal bleeding/prolapse (Figure 5a). Similar to the other TGFBR-deficient models, cATG mice also presented with reduced p21 expression and increased proliferation, indicative of a loss of TGFβ-induced cell cycle arrest (Figure 5b, 5c).

### TGFBR-deficiency increases tumor associated inflammation in conditional APC Δ468 driven colon carcinogenesis

**Figure 6:**
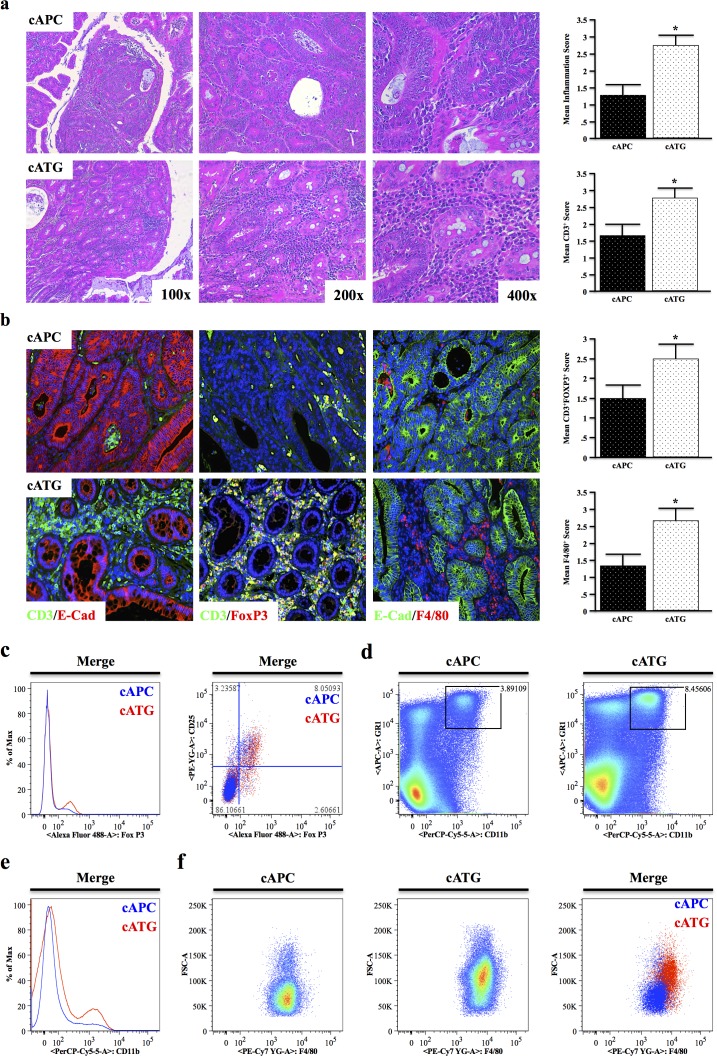
TGFBR-Deficiency Increases Tumor Associated Inflammation in Conditional APC Δ468 Driven Colon Carcinogenesis **a.,b.** Tissue sections from four-month old cAPC and cATG mice were stained with H&E and tumor infiltrating leukocyte infiltration scored by two investigators. Sections were also dual-stained with CD3/E-Cadherin to identify T cells, CD3/E-Cadherin to label regulatory T cells (Tregs), or F4/80/E-Cadherin to identify macrophages and scored similarly. **c.** The presence of live CD4+FoxP3+CD25+ Tregs in the mesenteric lymph nodes of cAPC and cATG mice was detected via flow cytometry. **d.,e.** Spleens were harvested from nongenic cAPC and cATG animals, and analyzed for expression of the myeloid marker CD11b and GR1, a marker of most inflammatory granulocytes, showing a pronounced increase in CD11b+GR1+ cells in cATG mice. **f.** Gating to GR1+CD11b+ populations in cAPC and cATG mice revealed increased F4/80+ splenic macrophages in cATG. (*, P < 0.05. N = 4 per group).

At four months of age, 8/8 cATG mice presented with large tubular adenomas compared to only 2/8 cAPC mice. Similar to the previous models, cATG mice displayed significantly higher leukocyte infiltration, particularly with respect to CD3^+^ T lymphocytes, which localized predominantly to the stroma (Figure 6a, 6b). However, T cells in cATG were highly FoxP3 positive (Figure 6b), despite CD4 cells from TG animals resisting TGFβ-induced FoxP3 up-regulation ex vivo [18]. Also consistent with an increased inflammatory burden, cATG mice had increased macrophage infiltration, also localizing to the stroma (Figure 6b).

To further assess changes in inflammatory cell populations, spleen and mesenteric lymph nodes were harvested from seven-month cAPC and cATG mice and subjected to flow cytometry. Consistent with our previous observations, in the lymph node CD4^+^ cells had increased FoxP3 expression, particularly in cells also expressing CD25 (Figure 6c). In the spleen, cATG mice displayed a similar increase in CD11b^+^GR1^Hi^ cells (Figure 6d), as well as an overall increase in myeloid cells (Figure 6e). Finally, gating to CD11b^+^GR1^Hi^ cells revealed these cells were also highly positive for F4/80 (Figure 6f), further suggesting that deficiency of TGFβ signaling increases inflammation in colon cancer.

## DISCUSSION

A multitude of TGFβ signaling inhibitors are currently under investigation as a potential cancer therapy, and are showing promise in other gastrointestinal cancers. Therefore, in this report, we sought to assess the effects of reduced TGFβ signaling on colon cancer development, in hopes of better risk-stratifying patients for potential treatment. However, our data demonstrated that genetic ablation of TGFβ signaling, either in epithelial cells or systemically, accelerates colon cancer development and increases tumor-associated inflammation in vivo.

The dual roles of TGFβ in carcinogenesis, while still poorly understood, are becoming increasingly recognized. TGFβ may inhibit proliferation and induce apoptosis in both normal intestinal epithelium and well-differentiated cancer cells. However, in many advanced tumors, TGFβ promotes invasion and metastasis [3, 7, 19–21]. In colon cancer, there appears to be a stepwise process involving the loss of growth suppressive TGFβ signaling, and eventual dominance of pro-migratory TGFβ signaling in advanced cancers. Several studies support this notion: TGFBR1 mutations increase the overall risk of colorectal cancer [22] and loss of SMAD4 in colon cancer results in worse survival and earlier recurrence [23–25]. However, in advanced cancer, TGFβ1 is often overexpressed [8] and correlates with worse disease [26]. Similarly, TGFBR2 mutations are particularly common in MSI colon cancers, which generally have an improved prognosis [27].

One theme that is currently under investigation to explain these events is the effects of TGFβ on the tumor microenvironment. In addition to affecting epithelial cells, TGFβ signaling can regulate tumor progression through its effects on inflammatory cells. Inconsistent with our findings, one clinical study examining an Eastern European cohort of genomically undefined colon and rectal cancers found that increased levels of cytoplasmic TGFβ1 and loss of SMAD4 were associated with reduced tumor-infiltrating macrophages. Furthermore, this study suggests that low macrophage density is associated with poor prognosis [28]. This has been observed in other patent cohorts [29], which also suggest that macrophages may have tumor suppressive functions.

This seemingly contradicts our findings, where both loss of SMAD4 and overexpression of TGFβ1 in the tumor microenvironment correlated with increased myeloid cell infiltration. However, it should be noted that our patient study used the pan-myeloid marker CD11b, which is not specific to macrophages. Furthermore, the roles of macrophages and other immune cells are highly varied in colon cancer, contingent on the tumor microenvironment [30], and possibly altered in genetic subtypes of colon cancer, which were not taken into account in any of these studies, ours included.

Also, in patients, there is considerable evidence that tumor-associated inflammation drives colon cancer progression and may have strong prognostic relevance [31]. It is well known that patients presenting with inflammatory bowel disease (IBD) have a 10-fold greater risk of colorectal cancer [32], suggesting that increased inflammation signaling may also enhance carcinogenesis. Moreover, murine models deficient of the anti-inflammatory cytokine IL10 develop pronounced colitis, are predisposed to inflammation associated colorectal cancer, and display overexpression of TGFβ1 [33].

To this effect, the effects of TGFβ on immune function are only recently becoming clear. Mouse models with knockout of TGFβ1 develop rapid and severe inflammatory lesions in multiple organs and a rapid wasting syndrome, leading to in death at 3 to 5 weeks of age [34]. As mentioned, TGFβ is a critical regulator of macrophage function, and mice lacking TGFBR2 in the bone marrow present with a lethal inflammatory phenotype [14]. Furthermore, depletion of TGFBR2 specifically in CD4^+^ T cells leads to early onset lethal autoimmune disease, further substantiating the anti-inflammatory role of TGFβ [34]. However, mice with ablation of TGFBR2 in both CD4 and CD8 cells under control of the distal Lck promoter did not show signs of autoimmunity [35]. Our study further reinforces the extremely complicated effects of TGFβ on the inflammatory response in the intestinal tract, and the consequence of TGFβ inhibition in specific leukocyte subsets is of critical importance when designing therapies targeting immune modulators such as TGFβ.

Combined, these studies further underscore the dire need to dissect the many roles of TGFβ and immune cells alike in colon cancer incidence and progression prior to targeting either for therapy. In our study, we found that TGFβ signaling deficiency accelerated mutant APC-induced models of intestinal polyposis, resulting in high levels of inflammatory cytokines, particularly IL8. Loss of TGFβ signaling also led to enhanced tumor-associated inflammation, increased tumor burden, and increased mortality. Our model of epithelial TGFβ signaling deficiency (ATE) is physiologically relevant, and mimics somatic mutations akin to those in patients with colon tumors [36]. These results may offer a partial explanation for the increased inflammation observed in many colon cancer patients, and similar observations have been made in other models of epithelial TGFBR deficiency [37].

However, as discussed, the effects of TGFβ in the tumor microenvironment are highly varied, contextually promoting and suppressing tumor development and associated inflammation. Therefore, we generated a model of global TGFβ signaling deficiency (ATG), which is more relevant to a systemically administered therapy targeting the TGFβ pathway. When compared to APC mice with intact TGFβ signaling, these mice still displayed accelerated tumor development, yet not to the extent of ATE mice with strictly epithelial TGFBR deficiency. These mice also displayed increased tumor associated inflammation compared to APC controls, yet also not to the extent of ATE mice. Interestingly, ATG mice had reduced IL17 production, suggesting reduced activity of the pro-inflammatory Th17 population [38], offering one possible explanation for these diametrically opposed findings. Finally, these results were corroborated in a more clinically relevant model of APC mutation (cAPC), which displayed nearly identical inflammatory phenotypes, further substantiating the TGFβ pathway as anti-inflammatory in colon cancer.

Thus, while targeting the tumor-promoting aspects of TGFβ is appealing in colon cancer given its pro-metastatic function in advanced disease, our data suggests that administering systemic TGFBR-inhibitors may further accelerate carcinogenesis in many, if not all, colon cancer patients. Therefore, further study is required before translating TGFBR therapy to the management of late-stage colorectal cancers. We conclude that caution must be exercised when designing clinical trials with TGFβ or related signaling inhibitors, as we predict a worse outcome due to overwhelming inflammatory responses.

## MATERIALS AND METHODS

### Cell lines and co-cultures

Human colon fibroblasts (CCD18) and well-differentiated human colon cancer cells (FET) were cultured in Dulbecco's Modified Eagle Medium (DMEM) and DMEM/F12 media respectively, each supplemented with 10% heat-inactivated fetal bovine serum (FBS), penicillin (100 units/mL), and streptomycin (100 μg/mL). All cells were cultured in a 37°C incubator with 5% CO_2_.

CCD18 cells were purchased from the American Type Culture Collection (ATCC) and used less than six months from purchase, and kept under passage 8. Early passage FET cells were provided by the lab that originally isolated these cells. All cell lines in the laboratory are tested for mycoplasma every six momths via LookOut Mycoplasma PCR Detection Kit (Sigma Aldrich, St. Louis, MO) and, if positive, treated with w/ Plasmocin (InvivoGen, SanDiego, CA) until mycoplasma could not be detected with the aforementioned kit.

Co-cultures were established by seeding epithelial cells in the bottom of six well plates, and tissue-corresponding stromal cell lines in transwell inserts in separate plates. Cells were allowed to adhere in their own media for 24 hours, then the stroma containing transwell inserts were added to the 6 well plates containing the epithelial monolayers. Cells were given fresh media, allowed to acclimate for 24 hours, and starved of serum overnight prior to TGFβ treatment.

### Mice

APC^Δ468^ (APC) mice were generated, as were cohorts of nongenic (control), MT-TGFBR2^DN^ (TE) or Tgfbr1^+/−^ (TG) (N = 3 per group). These mice were crossed to generate APC-TE (ATE), APC-TG (ATG) mice, that were euthanized at four months (N = 8 per group). Ts4-Cre mice were also generated and crossed to APC^fl/fl^ animals to form cAPC. cAPC mice were also crossed with TG animals to form cATG mice. These mice were euthanized at four months (N = 8 per group). For euthanasia, animals were anesthetized with xylazine/ketamine until unresponsive to toe tap and/or agonal breathing. Thoracotomy served as the primary form of euthanasia and exsanguination the secondary.

### Histology, immunohistochemistry/immunofluorescence, and tissue scoring

Age-matched control, APC, ATE, ATG, cAPC, and cATG mice were euthanized and subjected to pathological examination of the colon, small bowel, spleen, liver, lung, pancreas, and brain. Tissues were fixed in 10% formalin, paraffin-embedded, and sections at 4μm interval were cut from each tissue, and stained with H&E, Chloracetate Esterase (CAE), or via immunohistochemistry (IHC)/immunofluorescence (IF).

For IHC, slides were deparaffinized by xylenes and rehydrated by ethanol gradient, then heated in a pressure cooker using DAKO retrieval buffer. Endogenous peroxidases were quenched in 3% hydrogen peroxide in methanol for 30 min. Tissues were blocked with 0.5% BSA in PBS for 30 min and incubated with primary antibodies against pSMAD2 (Cell Signaling, Danvers, MA), p21 (Santa Cruz Biotech, Dallas, TX) at 1:50-1:200 overnight at 4°C. Slides were developed using either Streptavidin or secondary antibodies, followed by DAB substrate/buffer (DAKO, Carpinteria, CA).

For IF, slides were heated via pressure cooker in DAKO retrieval buffer and tissues blocked with 0.5% BSA in PBS for 1 hour at room temperature. Sections were exposed to primary antibodies against CD, PCNA (Santa Cruz), CD11b, FoxP3, TGFβ1, αSMA, GranzymeB (abcam, Cambridge, MA), E-Cadherin, Vimentin (Cell Signaling), and F4/80 (eBioSci, San Diego, CA) at 1:50-1:200 overnight at 4°C. Slides were developed using alexaflour 488 or 594 conjugated secondary antibodies (1:200-1:500, abcam), mounted in DAPI containing media (Santa Cruz), exposed to DAPI, FITC, and Texas Red filters, and images superimposed. All staining was compared to isotype and secondary antibody controls, which were negative for all antibody types used, and high magnification used to confirm mononuclear cells when assessing leukocyte populations.

For CAE staining, slides were deparaffinized by xylenes and rehydrated by ethanol gradient. New Fuchsin, 4% Sodium Nitrite, Phosphate Buffer, and Naphthol AS-D Chloroacetate were combined over ice and applied to tissue sections for 20 minutes. Slides were rinsed and counterstained with hematoxylin before mounting. All tissue counts and scores were performed by two blinded investigators. For any contradicting scores, a third investigator was consulted.

Staining intensity was determined by two blinded and independent investigators. Tissues with undetectable expression were scored as 0, and tissues with strong, ubiquitous expression scored 3+. For sections with intermediate staining, scores of 1-2+ were assigned by at least two blinded investigators based on variance from 0 and 3+.

### Flow cytometry

Mouse spleen, thymus, and mesenteric lymph nodes were isolated, ruptured, washed in PBS, and contents filtered. Two million cells were seeded into a round-bottom 96-well plate, washed in PBS, and stained with anti-Ly6G/GR1-APC, CD11b-PerCP-Cy5.5, and F4/80-PE-Cy7 (Biolegend, San Diego, CA), and an Alive/Dead kit (Invitrogen, Grand Island, NY) at 1:200-1:1500 in PBS at room temperature for 20 minutes. Cells were then fixed with 1% PFA in PBS for 10 minutes at room temperature. Cells were analyzed with a BD Fortessa Cytometer. All images correspond to live, single cells and are representative of 100,000 events unless otherwise stated.

### ELISA/multiplex assay

Animals were anesthetized with xylazine/ketamine and peripheral blood drawn directly from the heart into a heparin-containing syringe. Blood was centrifuged at 1,000 RCF to isolate serum. Sera were then subject to TGFβ1 ELISAs (eBioSci) or LegendPlex (BioLegend), both used per manufacturer specification and analyzed by plate reader or flow cytometry respectively.

### Statistical analysis

Data were analyzed by two-way ANOVA and fit to a general linear model in Minitab16, the validity of which was tested by adherence to the normality assumption and the fitted plot of the residuals. Results were arranged by the Tukey method, and were considered significant at p < 0.05. In vitro results are presented as ± S.D., and in vivo/clinical results are presented as mean ± S.E.M unless otherwise noted.

### Abbreviations

Transforming Growth Factor-β (TGFβ), Transforming Growth Factor β Receptor (TGFBR), Tumor Microenvironment (TME), Gastrointestinal (GI) Epithelial TGFBR-deficient (TE), Epithelial TGFBR-deficient (TG), APC^Δ468^ (APC), APC-TE (ATE), APC-TG (ATG), Conditional APC mutant (cAPC), cAPC-TG (cATG).

## SUPPLEMENTARY MATERIALS FIGURES AND TABLES


