# Supplementary material for: Loss of TGFβ signaling promotes colon cancer progression and tumor-associated inflammation
Source: Oncotarget. 2016 Jun 4;8(3):3826–39. doi: 10.18632/oncotarget.9830 (PMC5354798; doi:10.18632/oncotarget.9830)
Supplement: Supplementary file 1 [file oncotarget-08-3826-s001.pdf]

Loss of TGFβ signaling promotes colon cancer progression and tumor-associated inflammation

Supplementary Material

Table S1. Staining Results Arranged by Specimen

Patient data from Figure 1 are presented in the above table, arranged by score for SMAD4 intensity, number of $CD11b^+$ cells per 10x field, and TGFβ1 score.

Patient ID	SMAD4	CD11b	TGFβ1
353031	1	354	3
353032	2	71	2
353033	2	97	3
353034	3	94	3
353035	2	13	3
353036	3	112	2
353037	3	33	0
353039	0	226	3
353041	2	40	3
353042	0	843	3
353043	1	121	2
353045	3	51	3
353046	3	38	2
353047	1	262	1
353048	3	47	3
353050	3	27	2
353051	1	184	3
353052	3	73	3
353054	3	146	1

Table S2. Scoring Criteria for Human and Mouse Tissues

All stains were analyzed by two blinded investigators and assigned scores based on the following criteria.

Score	0	1+	2+	3+
**Criteria**	Majority of tissue showing negative staining indistinguishable from negative and isotype controls.	Sporadic or generally weak staining, yet more robust than negative and isotype controls.	Uniform modest staining and/or isolated areas of strong staining.	Uniform strong staining throughout the sample.

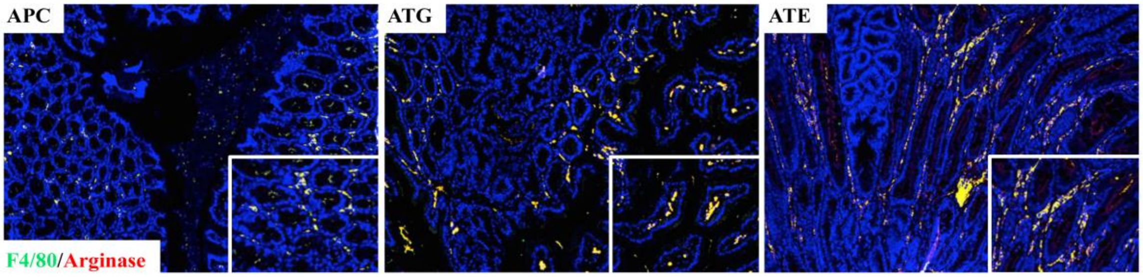

Figure S1. Epithelial and Systemic TGFBR-Deficiency Does Not Alter Macrophage Polarization *in Vivo*

Colon tissue from age matched APC, ATG, and ATE mice was stained for F4/80, a murine macrophage marker, and arginase, a marker of M2 polarization. No significant difference was observed across any group.

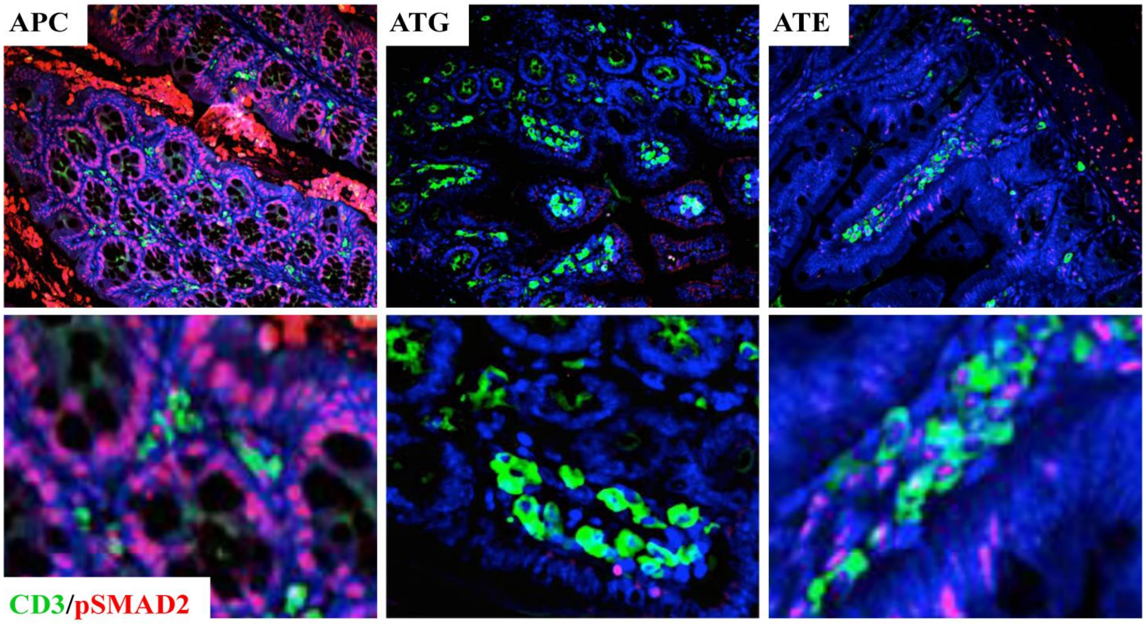

Figure S2. The MT-TGFBR2^{DN} Transgene Does Not Directly Affect the T Cell Compartment

Colon tissue from APC, ATG, and ATE mice was next dual stained for CD3 and pSMAD2, indicating that ATE mice still have intact TGFβ signaling in T cells.

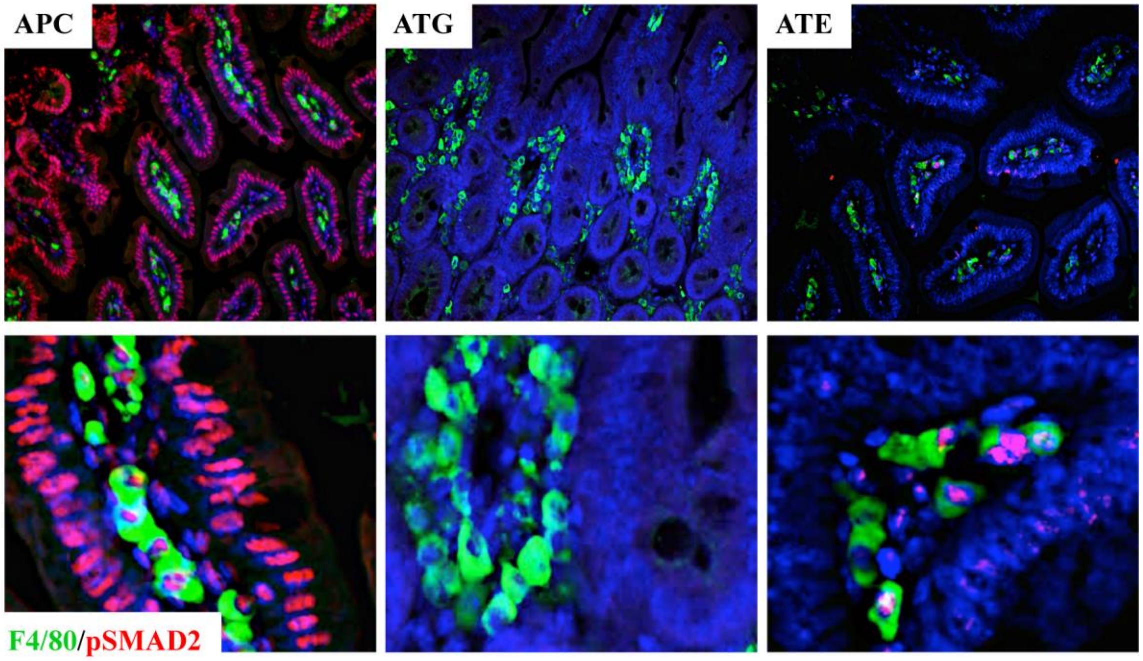

Figure S3. The MT-TGFBR2^{DN} Transgene Does Not Directly Affect Macrophages

Colon tissue from APC, ATG, and ATE mice was dual stained for CD3 and pSMAD2, affirming that ATE mice still have intact TGFβ signaling in macrophages.

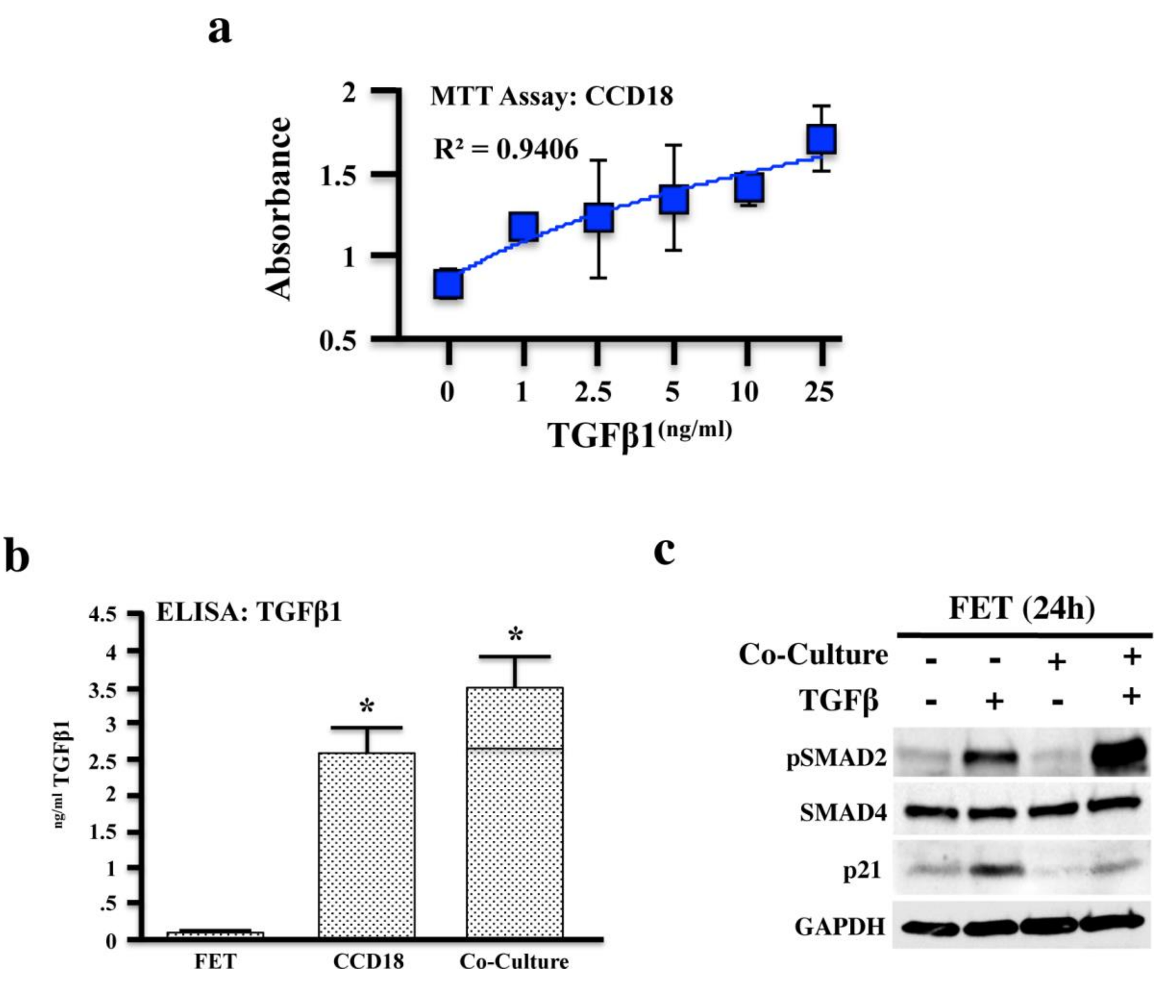

Figure S4. TGFβ Overexpression is Mediated by the Colon Stroma

(a) CCD18 colon fibroblasts were serum-starved and pulsed with 0-25 ng/ml TGFβ1. After 24 hours, an MTT assay demonstrated increased proliferation of colon fibroblasts in a dose dependent manner to TGFβ1. **(b)** Co-cultures of FET human colon cancer cells and CCD18 colon fibroblasts were established and showed significantly elevated TGFβ1 in the culture media when compared to isolated cultures of either cell line. **(c)** FET/CCD18 co-cultures pulsed with exogenous TGFβ1 and downstream signaling evaluated by western blot.
